# The multidisciplinary strategy to severe neck contracture and complex post-tracheostomy tracheal stenosis: A case report

**DOI:** 10.1097/MD.0000000000047122

**Published:** 2026-01-16

**Authors:** Jinyue Jiang, Yang Bai, Jian Tang, Xiao Shen, Jia Zhou, Yishi Li

**Affiliations:** a Department of Respiratory and Critical Care Medicine, The First Affiliated Hospital of Chongqing Medical University, Chongqing, China; b Department of Respiratory and Critical Care Medicine, Neijiang Dongxing District People’s Hospital, Sichuan, China; c Department of Burn and Plastic Surgery, The First Affiliated Hospital of Chongqing Medical University, Chongqing, China.

**Keywords:** interventional pulmonology, postburn neck contracture, post-tracheostomy tracheal stenosis, silicone stent, supraclavicular flap

## Abstract

**Rationale:**

Complex post-tracheostomy tracheal stenosis (PTTS), characterized by cartilage destruction and tracheomalacia, is a severe, rare complication in burn patients. Concomitant severe postburn neck contractures impair airway access, rendering conventional treatments (e.g., tracheal resection and endoscopic interventions) challenging and necessitating multidisciplinary strategies.

**Patient concerns:**

A 49-year-old male with a 2-year history of banana oil-induced burns presented with exertional dyspnea (modified Medical Research Council Grade 3). He had failed prior endoscopic interventions and refused surgical tracheal resection. Severe neck contractures limited mobility and airway access.

**Diagnoses:**

Complex PTTS (Cotton-Myer Grade III, 6-mm luminal narrowing, cartilage destruction, and tracheomalacia) confirmed via computed tomography and bronchoscopy; severe postburn neck contracture (microstomia, fixed flexion deformity) on physical exam.

**Interventions:**

A two-stage multidisciplinary approach was implemented: neck contracture release with a pre-expanded 18 × 10 cm supraclavicular artery perforator flap; rigid bronchoscopy-guided placement of a 10 × 50 mm hourglass-shaped silicone stent.

**Outcomes:**

Postoperatively, the patient regained normal mastication, phonation, and respiration by the fourth week. At the 4-year follow-up, he maintained tracheal patency (modified Medical Research Council Grade 1), had restored neck function, and experienced no stent complications. He declined further surveillance.

**Lessons:**

Multidisciplinary collaboration is critical for managing complex PTTS with severe postburn neck contractures in patients unsuitable for surgery. Pre-expanded supraclavicular flaps enable safe airway interventions, while hourglass stents effectively maintain patency. Prospective studies are needed to standardize such protocols.

## 1. Introduction

Tracheal stenosis, though rare in burn patients, is a serious complication that typically results from direct inhalation injuries or prolonged mechanical ventilation.^[1]^ Post-tracheostomy tracheal stenosis (PTTS), prevalent among patients with extended tracheostomy intubation, induces granulation tissue formation at the stoma site and may progress to structural collapse due to cartilage destruction and tracheomalacia (complex PTTS).^[2,3]^ While tracheal resection with end-to-end anastomosis demonstrates high success rates (up to 95%) for benign tracheal stenosis,^[4–6]^ these procedures carry inherent risks of perioperative mortality, especially in complex PTTS cases.^[7]^ For patients deemed high-risk for surgery or those declining invasive repair, silicone stents serve as a clinically validated nonsurgical alternative.^[8,9]^

Concurrently, postburn neck contracture, a frequent sequela of neck burns manifesting as microstomia (small mouth), fixed flexion deformities, and difficult airway anatomy, significantly complicates both airway management and aesthetic rehabilitation.^[10,11]^ Severe neck contractures introduce technical barriers to rigid bronchoscopy insertion and silicone stent placement, as quantified by the Simplified Airway Risk Index.^[12]^ The co-occurrence of severe neck contracture and complex PTTS necessitates a multidisciplinary management strategy. This case report from the First Affiliated Hospital of Chongqing Medical University details such a clinical scenario, adhering to The Surgical CAse REport guidelines.^[13]^

## 2. Case presentation

**Figure 1.**
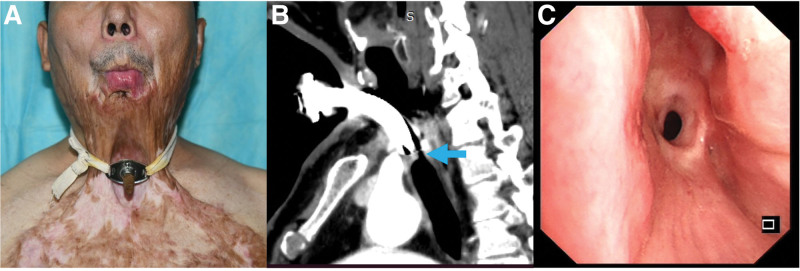
Clinical and diagnostic features of severe postburn neck contracture and complex tracheal stenosis. (A) Microstomia (small mouth) and fixed cervical flexion deformity resulting from severe postburn neck contracture. (B) Sagittal chest computed tomography image demonstrating severe tracheal stenosis (blue arrow) below the tracheostomy stoma site, with the narrowest diameter measuring 6 mm. (C) Bronchoscopy view confirming complex post-tracheostomy tracheal stenosis (bottleneck sign) below the stoma site.

A 49-year-old man with severe postburn neck contracture secondary to banana oil exposure involving the face, neck, chest, and hands was referred to our department. Two years prior, he had undergone the tracheotomy and adjuvant therapies at a local hospital. He presented with a 6-month history of exertional dyspnea (modified Medical Research Council Grade 3), accompanied by cough and sputum production, but without dysphagia or hypersalivation. The patient had no significant comorbidities or family medical history. Physical examination revealed severe postburn neck contracture extending from the lower lip to the anterior chest, causing microstomia, loss of the cervicomental angle, forced anterior head posture, and fixed flexion deformity (Fig. 1A). A tracheotomy tube was in situ with audible stridor over the anterior neck. Laboratory findings were unremarkable. Chest computed tomography demonstrated complex PTTS with cartilage destruction distal to the tracheotomy tube, revealing a narrowest luminal diameter of 6 mm (Fig. 1B). Bronchoscopy confirmed an endoluminal obstruction (Cotton-Myer Grade III)^[14]^ exceeding 1 cm in length, with associated scar tissue, granulation hyperplasia, and tracheomalacia (bottleneck sign; Fig. 1C). The patient prioritized tracheotomy decannulation and functional recovery.

Despite multiple bronchoscopic interventions, including electrocautery, radical scar incision, and high-pressure balloon dilatation, the patient failed to tolerate tracheotomy tube capping. Repeat bronchoscopy revealed recurrent stenosis secondary to cartilage destruction and tracheomalacia. The patient declined surgical resection with end-to-end anastomosis due to concerns regarding potential complications, prolonged recovery, and uncertain outcomes. Instead, he opted for less invasive alternatives, such as silicone stent placement. However, severe postburn neck contracture precluded rigid bronchoscopy insertion, as quantified by the Simplified Airway Risk Index (mouth opening < 4 cm, thyromental distance < 6 cm, Mallampati classification Ⅳ, neck movement < 80°, underbite, body weight < 90 kg, and prior difficult intubation). A multidisciplinary team comprising anesthesiologists, pulmonologists, otolaryngologists, and plastic surgeons devised a two-stage procedure: postburn neck contracture release and reconstruction with a pre-expanded supraclavicular artery perforator flap, followed by an hourglass-shaped silicone stent placement.

**Figure 2.**
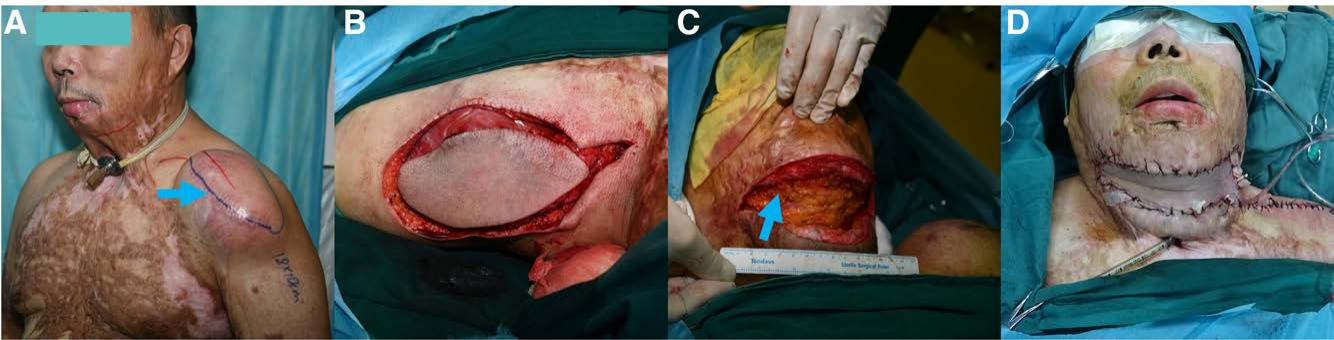
Surgical reconstruction using a pre-expanded supraclavicular artery perforator flap. (A) Preoperative marking of the pre-expanded supraclavicular artery perforator flap (blue arrow) on the left shoulder. (B) Complete harvest of the flap in the subfascial plane. (C) Excision of cervical contracture (blue arrow) guided by the size of the harvested flap to obtain maximum neck extension. (D) Flap insertion into the defect, with subcutaneous closure using bio-absorbable suture and skin closure with nonabsorbable suture. A plaster splint was applied, and the donor site was closed after flap insertion.

**Figure 3.**
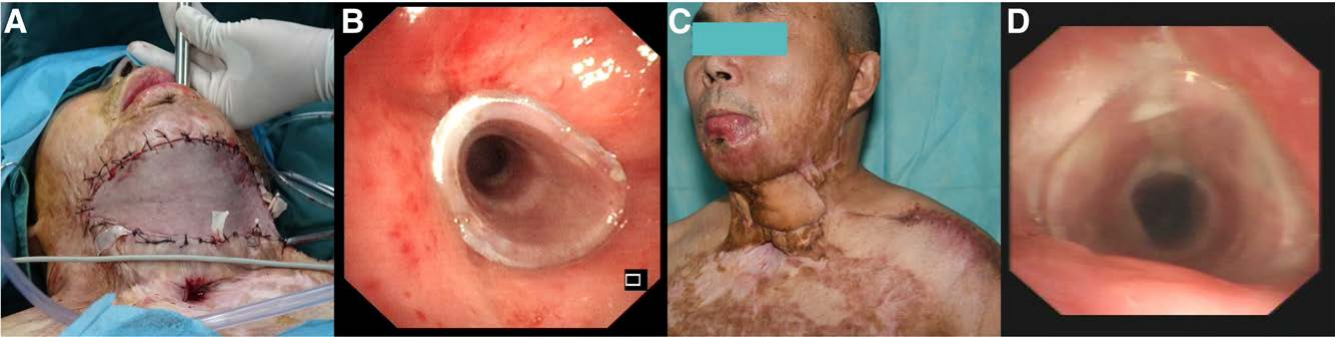
Postoperative outcomes and stent placement. (A) Insertion of the rigid bronchoscope immediately following the surgical reconstruction of the postburn neck contracture. (B) Successful placement of an hourglass-shaped silicone stent at the site of complex tracheal stenosis. (C) Four weeks postoperatively, the patient demonstrated complete neck extension, with the flap and graft fully healed over the neck and shoulder. (D) Bronchoscopic view showing the hourglass-shaped silicone stent in place, with no migration or granulation during follow-up.

Under local anesthesia, a silicone tissue expander was implanted subcutaneously at the left shoulder to generate an 18 × 10-cm flap (Fig. 2A). The pre-expanded supraclavicular artery perforator flap was harvested using established techniques (Fig. 2B).^[15,16]^ Contracture release created a 14 × 10-cm defect (Fig. 2C), achieving sufficient neck extension for rigid bronchoscopy insertion under general anesthesia with intubation via tracheotomy. The defect was fully covered by the flap (Fig. 2D). Post-reconstruction, a rigid bronchoscope was successfully inserted into the trachea (Fig. 3A), and an hourglass-shaped silicone stent (NOVATECH, Aubagne, France), with an inner diameter of 10 mm and a length of 50 mm, was placed after electrocautery and balloon dilatation (Fig. 3B). The patient recovered uneventfully, regaining normal mastication, phonation, and respiration by postoperative week 4 (Fig. 3C). Donor-site shoulder mobility remained intact. A 7-month bronchoscopy confirmed stent stability without complications (Fig. 3D). During a 4-year follow-up through telephone interviews, the patient reported occasional thick sputum but no dyspnea (modified Medical Research Council Grade 1) and declined further bronchoscopic evaluations.

## 3. Discussion

Tracheal stenosis, a rare yet debilitating complication in burn survivors, primarily originates from direct inhalation injury or prolonged tracheostomy-dependent ventilation. The incidence of tracheal stenosis among burn patients was estimated at 0.37%, with inhalation injuries accounting for 71% of cases and tracheostomy-related stenosis comprising the remainder.^[17]^ Prolonged tracheostomy predisposes to the formation of tracheal stenosis, tracheomalacia, and tracheoesophageal fistula.^[18]^ PTTS can be divided into either simple (web-like) or complex (bottleneck-like) stenosis, with the latter characterized by cartilage destruction and tracheomalacia through imaging and bronchoscopic evaluation.^[19,20]^ The development of PTTS mainly involves abnormal wound healing, which is characterized by aberrant granulation tissue proliferation at the stoma sites. This process is further exacerbated by mechanical factors such as shear forces from the tube and pressure caused by the ventilator circuit, ultimately leading to cartilage necrosis and destruction.^[21,22]^ In this case, complex PTTS manifested as a circumferential, bottleneck-shaped obstruction (>1 cm in length) refractory to endoscopic therapies (mechanical debridement, electrocautery, and balloon dilatation). While tracheal resection with end-to-end anastomosis remains the gold standard for benign tracheal stenosis (reported success rates of 85–95%^[4–6]^), its applicability in complex PTTS is limited by perioperative risks and substantial resource demands.^[7]^ Silicone stenting thus serves as a critical alternative for high-risk patients or those declining surgery.^[8,9]^

Postburn neck contractures, affecting 8% of patients with second-degree neck burns, present profound functional impairment and aesthetic deformities.^[10,23]^ These contractures demonstrate higher prevalence and severity in developing regions, attributable to extensive open-flame exposures and inadequate acute/rehabilitative burn care.^[24]^ We reported a case of severe postburn neck contracture with microstomia, fixed flexion deformity, restricted atlanto-occipital joint mobility, and an anticipated difficult airway, quantified by a Simplified Airway Risk Index score of 10.^[12]^ Surgical intervention focused on 3-dimensional contracture release through scar tissue excision, cervical mobility restoration, and anatomical contour reconstruction, ultimately allowing the insertion of a rigid bronchoscope. The reconstructive strategy employed a pre-expanded supraclavicular artery perforator flap, a technique increasingly favored in postburn neck reconstruction due to its regional tissue compatibility (color/texture match), reliable vascularity, and capacity for substantial defect coverage without microvascular anastomosis.^[25–27]^ The protocols enhanced flap dimensions while minimizing donor-site morbidity through controlled tissue expansion, achieving complete coverage of the extensive cervical defect in this case.^[28–30]^

Post-reconstruction airway management involved placing an hourglass-shaped silicone stent to address the complex PTTS. This stent design incorporates biomechanical stability through differential radial forces – increased distal/proximal expansile forces coupled with a constricted mid-portion – effectively anchoring the device within the subglottic trachea while reducing granulation tissue proliferation.^[31]^ The entire procedure was performed by a multidisciplinary team, underscoring the importance of a collaborative approach in managing the co-occurrence of severe postburn neck contracture and complex PTTS.

While demonstrating a proof-of-concept for this combined approach, study limitations include inherent constraints of single-case reporting and patient-driven treatment preferences, excluding anastomotic options. These factors highlight the need for developing evidence-based protocols through prospective multicenter studies, particularly for optimizing decision-making in patient-specific anatomical constraints.

## 4. Conclusion

This case exemplifies the critical role of a multidisciplinary strategy in managing the co-occurrence of severe postburn neck contractures and complex PTTS. Pre-expanded supraclavicular artery perforator flaps provide anatomically appropriate neck reconstruction while enabling subsequent airway interventions. Hourglass-shaped stenting emerges as an important nonsurgical alternative for complex PTTS in anatomically compromised patients. Future studies should prioritize establishing standardized protocols for managing such cases with severe postburn neck contractures and complex PTTS.

## Author contributions

**Conceptualization:** Yang Bai, Yishi Li.

**Funding acquisition:** Jinyue Jiang, Yishi Li.

**Investigation:** Yang Bai, Jian Tang, Xiao Shen, Yishi Li.

**Project administration:** Xiao Shen, Yishi Li.

**Writing – original draft:** Jinyue Jiang, Yang Bai, Jian Tang, Jia Zhou, Yishi Li.

**Writing – review & editing:** Jinyue Jiang, Yang Bai, Jian Tang, Jia Zhou, Yishi Li.
